# Optimizing sowing patterns in winter wheat can reduce N_2_O emissions and improve grain yield and NUE by enhancing N uptake

**DOI:** 10.3389/fpls.2023.1176293

**Published:** 2023-05-31

**Authors:** Xiu Zhang, Manyu Liu, Feina Zheng, Yuanjie Dong, Yifan Hua, Jinpeng Chu, Mingrong He, Xinglong Dai

**Affiliations:** ^1^ College of Agronomy, Shandong Agricultural University, Tai’an, Shandong, China; ^2^ Agricultural and Rural Bureau of Mengyin County, Linyi, Shandong, China; ^3^ College of Resources and Environment, Shandong Agricultural University, Tai’an, Shandong, China; ^4^ College of Agriculture, Nanjing Agricultural University, Nanjing, Jiangsu, China

**Keywords:** N rate, wide belt sowing, N_2_O emissions, grain yield, plant N uptake, soil inorganic N concentration, winter wheat (*Triticum aestivum* L.)

## Abstract

Increasing nitrogen (N) input is essential to satisfy the rising global wheat demand, but this increases nitrous oxide (N_2_O) emissions, thereby exacerbating global climate change. Higher yields accompanied by reduced N_2_O emissions are essential to synergistically reduce greenhouse warming and ensure global food security. In this study, we conducted a trial using two sowing patterns (conventional drilling sowing [CD] and wide belt sowing [WB], with seedling belt widths of 2–3 and 8–10 cm, respectively) with four N rates (0, 168, 240, and 312 kg ha^-1^, hereafter N0, N168, N240, and N312, respectively) during the 2019–2020 and 2020–2021 growing seasons. We investigated the impacts of growing season, sowing pattern, and N rate on N_2_O emissions, N_2_O emissions factors (EFs), global warming potential (GWP), yield-scaled N_2_O emissions, grain yield, N use efficiency (NUE), plant N uptake and soil inorganic N concentrations at jointing, anthesis, and maturity. The results showed that sowing pattern and N rate interactions influenced the N_2_O emissions markedly. Compared to CD, WB significantly reduced cumulative N_2_O emissions, N_2_O EFs, GWP, and yield-scaled N_2_O emissions for N168, N240, and N312, with the largest reduction seen at N312. Furthermore, WB markedly improved plant N uptake and reduced soil inorganic N compared to CD at each N rate. Correlation analyses indicated that WB mitigated the N_2_O emissions at various N rates mainly through efficient N uptake and reduced soil inorganic N. The highest grain yield occurred under a combination of WB and N312, under which the yield-scaled N_2_O emissions were equal to the local management (sowing with CD at N240). In conclusion, WB sowing could synergistically decrease N_2_O emissions and obtain high grain yields and NUEs, especially at higher N rates.

## 1 Introduction

With an expanding world population, it is estimated that major cereal crops must increase by approximately 50% to meet the expected food demand by 2050 (vanDijk et al., 2021). However, limited land area for agriculture means that the only way to increase grain yield is to achieve a higher yield per unit of land area (Godfray et al., 2011). The nutrients compositions and quality of soil, especially nitrogen (N) nutrition, has a significant impact on crops productivity, thereby grain yield (Duan et al., 2019). It is foreseeable that more synthetic N fertilizers will be needed to meet the increasing grain yield demands of an increased global population (Kong et al., 2021).

Global warming caused by greenhouse gases is currently a research hotspot (Kim et al., 2013; Seth and Misra, 2014). It may exacerbate the occurrence of abiotic stresses, such as salt, drought, and so on (Zhu, 2016; Shultana et al., 2022). Application of exogenous matters, like inorganic N, may alleviate these abiotic stresses (Krapp, 2015; Agnihotri and Seth, 2016).

N_2_O, one of the most important greenhouse gases, is produced in soils, and approximately 60% of global N_2_O emissions originate from agriculture, mainly due to N fertilizer application to soils (Millar et al., 2018). N_2_O causes global warming, destroys the ozone layer, and increases ultraviolet radiation on the ground (IPCC, 2021). When seeking for the high yield or alleviating these abiotic stress in crop production, the application of N fertilizer may increase N_2_O emissions, because the soil NH_4_^+^ and NO_3_^−^ concentrations increase (Millar et al., 2018; Takeda et al., 2021), both of which are the substrates of nitrification and denitrification processes in soil and closely related to N_2_O emissions (Subbarao et al., 2017; Zhang et al., 2019).

Therefore, increasing wheat yield while mitigating the cumulative N_2_O emissions caused by N fertilizer application is essential to ensure food security and slow global warming (Ying et al., 2019). The application of urease and nitrification inhibitors (Recio et al., 2019; Wang et al., 2021), control-released fertilization (Ji et al., 2012), and partial substitution of chemical N with manure (Kong et al., 2021; Zhang et al., 2021) could synergistically increase wheat yield and reduce N_2_O emissions. However, these measures will increase production costs.

Compared to conventional drilling sowing (CD), wide belt sowing (WB) is an optimized sowing pattern that increases the belt of wheat seedlings from 2–3 to 8–10 cm by altering the width of the furrow opener moldboard without increasing other costs. This improves spatial uniformity and reduces intraspecific competition within seedling belts (Liu et al., 2020; Lv et al., 2020), and results in enhanced water, N, radiation use efficiency, and grain yield of winter wheat (Li et al., 2015; Liu et al., 2020; Wang et al., 2022; Zheng et al., 2023). In particular, the enhanced ability to absorb N offers the possibility of reducing nitrous oxide emissions. However, there are insufficient data on how WB affects the N_2_O emissions of winter wheat.

Therefore, we hypothesized that sowing winter wheat as WB instead of CD with N fertilizer input would result in improved grain yield alongside reduced N_2_O emissions. This would be due to the reduced concentrations of inorganic N in the soil through enhanced N uptake. We evaluated the interaction between sowing pattern and the application of different N rates on grain yield and N_2_O emissions. To this end, we used two sowing patterns (CD and WB) at N rates of 0, 168, 240, and 312 kg ha^-1^ under field conditions. We also investigated the N uptake and soil inorganic N concentrations at jointing, anthesis, and maturity stages to elucidate the processes involved in decreasing N_2_O emissions using the optimized sowing pattern.

## 2 Materials and methods

### 2.1 Study site and growth conditions

During the 2019–2020 and 2020–2021 winter wheat growing seasons, field experiments were conducted in Dongwu Village (35°57’N, 117°03’E), Dawenkou, Daiyue District, Tai’an, Shandong Province, China. Summer maize was the previous crop grown at the study site, and all remaining straw was plowed into the field. The soil was characterized as a sandy loam (typic Cambisols; FAO, 2003) with a pH of 7.2. Before sowing wheat in 2019–2020, the total N, alkali-hydrolyzable N, available P_2_O_5_, available K_2_O, and organic matter in the top 20 cm of the soil were 1.11 g kg^-1^, 111.00 mg kg^-1^, 34.69 mg kg^-1^, 98.47 mg kg^-1^, and 16.70 g kg^-1^, respectively. Climatic data, including rainfall and temperature, are shown in **Figure S1**.

### 2.2 Experimental design

Seeds of two winter wheat cultivars, Tainong18 (T18) and Taimai198 (T198), were sown on 15 October 2019 and 17 October 2020 and harvested on 8 June 2020 and 10 June 2021, respectively. We used two sowing patterns (**Figure S2**; CD and WB) and four N rates (0, 168, 240, and 312 kg ha^-1^; hereafter, N0, N168, N240, and N312, respectively). The CD sowing pattern at N240 is widely used in local agricultural production. Treatments of each cultivar were arranged in a split-plot design with the N rate as the main plot and sowing pattern as the subplot (n = 4). The length and width of each subplot were 22.0 m and 3.0 m (12 rows spaced 25 cm apart), respectively. The basal/topdressing of N fertilizer (applied as urea, 46% N) in a 4:6 ratio, and the topdressing N was applied at jointing (**Table S1**). The crops were irrigated after sowing, at jointing and anthesis in both growing seasons, at a rate of 60 mm each time.

### 2.3 Measurement methods

#### 2.3.1 Grain yield, inorganic N concentrations in the soil, and plant N uptake

Grain yield was measured at maturity by manually cutting all spikes in 3.0 m^2^ rows in each plot as described by Li et al. (2015) and adjusted to 13% moisture content. The inorganic N concentrations in the soil and plant N uptake were measured according to Shi et al. (2012). We collected soil samples randomly from five locations in each plot before sowing, and at the jointing, anthesis, and maturity stages to estimate the inorganic N. Fifty single plants or stems were sampled at all three stages to determine the aboveground N accumulation (AGN). The plant N uptake during growth was calculated according to the AGN of the latter growth stage minus that of the previous growth stage. NUE, N uptake efficiency (UPE), and N utilization efficiency (UTE) were calculated according to Moll et al. (1982). The formulas for calculating these indexes are provided in the **Supplementary Material**.

#### 2.3.2 N_2_O emissions flux and calculation of N_2_O emissions-related indicators

We used the closed chamber-gas chromatography method to measure the N_2_O emissions flux, according to Lyu et al. (2019). In this study, the chambers included a chamber base of 54 cm length × 22 cm wide and 26 cm high with a 3 cm width water channel and a cover box of 56 cm × 24 cm × 90 cm. Gas was sampled daily for 5 days after base fertilization, and sampling frequency was reduced to once every 7 days for approximately 1 month after irrigation (after sowing) or rainfall > 20 mm and then three times a month until the next fertilization event. Gas samples were also measured daily following topdressing for 5 days and every 2 days after that for five times until irrigation at the anthesis stage. Gas samples were also measured after irrigation or rainfall > 20 mm every 7 days until maturity.

The N_2_O flux formula was calculated according to the adapted equation by Duan et al. (2019). The cumulative N_2_O emissions, fertilizer-induced N_2_O emissions factor (EF), and the yield-scaled N_2_O were computed referring to the equation in Huang et al. (2017). The N_2_O global warming potential (GWP) was calculated as the cumulative N_2_O emissions multiplied by 273, according to IPCC (2021). The formulas for calculating these indexes are provided in the **Supplementary Material**.

### 2.4 Statistical analysis

Analyses of variance and multiple comparisons were determined according to the least significant difference at 0.05 and a probability level with DPS 7.05 (Zhejiang University, Hangzhou, China). We used Microsoft Excel 2013 (Microsoft, Redmond, WA, USA) to create the tables and SigmaPlot 14.0 (Systat Software, San Jose, CA, USA) to generate the figures.

## 3 Results

### 3.1 Grain yield and NUE

The winter wheat grain yield was significantly affected by growing season, cultivars, N rates, sowing patterns, and the following interactions: growing season × N rate, cultivar× N rate, N rate × sowing pattern, and growing season × cultivar × N rate (**Table S2**). The grain yield increased significantly with an increased N rate from N0 to N312 (**Table 1**). WB significantly increased the grain yield at N168, N240, and N312 compared to CD, despite the increase showing a decreasing trend with the increased N rate. In 2019–2020, WB increased grain yields by 10.43%, 8.22%, and 5.44%, and by 10.31%, 7.67%, and 6.63% at N168, N240, and N312 for cultivars T18 and T198, respectively. In 2020–2021, the grain yields in WB increased by 9.18%, 8.24%, and 7.22%, and by 8.44%, 6.85%, and 5.74% for T18 and T198, respectively. WB at N312 showed the highest yield of all the treatments. Compared to local management (sowing with CD at N240), WB at N312 increased the grain yields of T18 and T198 by 8.03% and 13.53% in 2019–2020 and 15.16% and 13.06% in 2020–2021, respectively.

**Table 1:** Effects of sowing pattern and N rate on grain yield, N use efficiency (NUE), N uptake efficiency (UPE), and N utilization efficiency (UTE) of winter wheat.

Growing season	Cultivar	N rate (kg ha^-1^)	Sowing pattern	Grain yield	NUE	UPE	UTE
				(kg ha^-1^)	(kg kg^-1^)	(%)	(kg kg^-1^)
2019–2020	Tainong18	0	Conventional drilling	7254.94e	41.50b	88.86b	46.61a
			Wide belt	7754.59d	44.60a	91.88a	48.59a
		168	Conventional drilling	8412.29c	24.56d	72.05d	33.73b
			Wide belt	9289.77a	27.13c	80.59c	33.72b
		240	Conventional drilling	8656.55bc	20.92f	66.45f	31.49c
			Wide belt	9368.07a	22.60e	73.05d	30.95c
		312	Conventional drilling	8868.76b	18.25h	64.68g	28.36d
			Wide belt	9351.27a	19.25g	69.16e	28.32d
	Taimai198	0	Conventional drilling	7279.05f	41.80b	75.03b	55.94a
			Wide belt	7952.01e	45.66a	80.78a	57.17a
		168	Conventional drilling	8636.00d	25.24d	66.88d	37.84b
			Wide belt	9526.40c	27.84c	74.88b	37.10b
		240	Conventional drilling	9318.82c	22.50f	64.43e	34.78c
			Wide belt	10033.43b	24.23e	70.53c	34.36c
		312	Conventional drilling	9921.35b	20.41h	63.05e	32.23d
			Wide belt	10579.47a	21.76g	68.42d	31.96d
2020–2021	Tainong18	0	Conventional drilling	4562.38f	31.74c	77.96e	40.13a
			Wide belt	4808.72e	33.45b	84.28d	39.67a
		168	Conventional drilling	9049.36d	31.70c	91.40b	34.68b
			Wide belt	9880.08c	34.60a	101.53a	34.11b
		240	Conventional drilling	9784.59c	26.42e	79.71e	33.14c
			Wide belt	10591.14b	28.59d	87.46c	32.70c
		312	Conventional drilling	10512.10b	22.64g	72.96f	31.04d
			Wide belt	11270.99a	24.27f	79.15e	30.71d
	Taimai198	0	Conventional drilling	3956.82f	27.53f	75.40d	36.56ab
			Wide belt	4257.15e	29.61d	82.32c	36.18b
		168	Conventional drilling	9662.91d	33.84b	90.86b	37.27a
			Wide belt	10478.41c	36.70a	99.90a	36.74ab
		240	Conventional drilling	10648.62c	28.75e	84.12c	34.18c
			Wide belt	11378.58b	30.72c	90.80b	33.84c
		312	Conventional drilling	11386.15b	24.52h	77.44d	31.67d
			Wide belt	12039.84a	25.93g	82.89c	31.29d

Different letters within a column for the same season and cultivar indicate significant differences (P < 0.05).

The NUE of winter wheat was significantly influenced by growing season, cultivar, N rates, sowing patterns, and the interactions of growing season × cultivar, growing season × N rate, cultivar× N rate, N rate × sowing pattern, growing season × cultivar× N rate, and growing season × N rate × sowing pattern (**Table S2**). It significantly decreased with N rates increased from N168 to N312 (**Table 1**). WB significantly improved it at N168, N240, and N312 compared to CD, despite the extent of the increase showing a decreasing trend with the increased N rate. In 2019–2020, WB increased the NUE by 10.43%, 8.22%, and 5.44%, and by 10.31%, 7.67%, and 6.63% at N168, N240, and N312 for cultivars T18 and T198, respectively. In 2020–2021, the NUE in WB increased by 9.18%, 8.24%, and 7.22%, and by 8.44%, 6.85%, and 5.74% for T18 and T198, respectively.

Under each sowing pattern, the UPE and UTE decreased gradually with the increased N rate. Compared to CD, WB markedly increased the UPE at N168, N240, and N312. The UPE was increased by an average of 11.42%, 9.83%, and 7.71%, and by 10.88%, 8.52%, and 7.87%, at N168, N240, and N312 for the T18 and T198 cultivars, respectively, across two growing seasons. However, the UTE for either cultivar was not significantly different between CD and WB at each N rate.

### 3.2 N_2_O emissions

#### 3.2.1 N_2_O emissions dynamics

**Figure 1:**
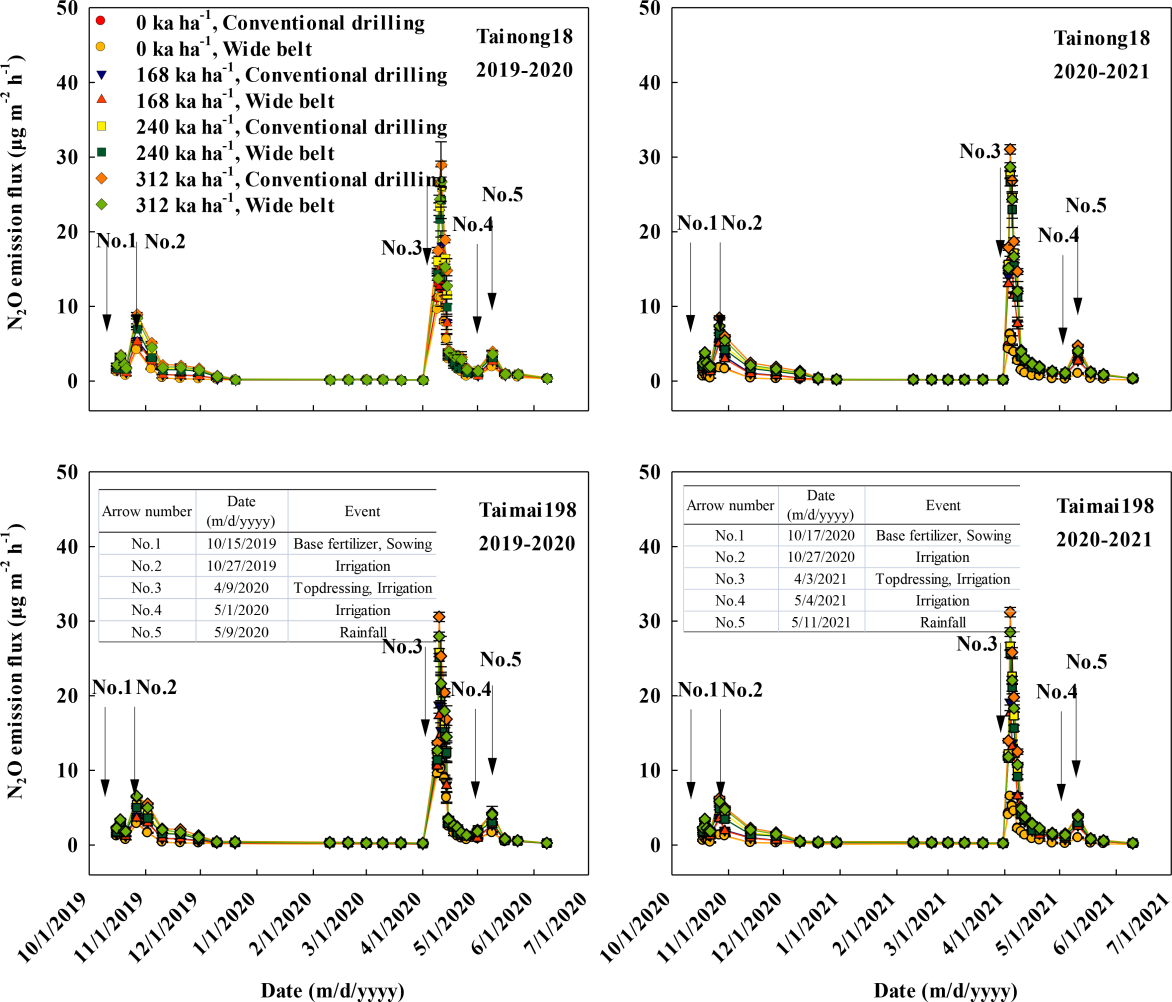
Effects of sowing pattern and N rate on the nitrous oxide (N_2_O) emissions fluxes of winter wheat. The rainfall events all exceeded 20 mm.

The dynamic changes in the N_2_O fluxes are presented in **Figure 1**. The N_2_O dynamics were considerably affected by the sowing patterns and N rates over the two growing seasons. N_2_O gradually increased with the increased N rate when sown using the same sowing pattern. Then it usually spiked after the input of basal fertilizer and topdressing fertilizer and after rainfall (> 20 mm) or irrigation (arrows numbered 1 to 5). The N_2_O emissions were higher in CD than in WB, and CD at N312 had the highest N_2_O flux.

N_2_O flux peaked after topdressing and was lower in WB at each N rate compared to CD. In 2019–2020, the highest fluxes were 12.98, 18.20, 26.00, and 29.04 μg m^-2^ h^-1^ and 12.75, 18.75, 25.77, and 30.56 μg m^-2^ h^-1^ for N0, N168, N240, and N312 of the T18 and T198 cultivars, respectively, when sown in CD. However, when sown in WB, they were 11.20, 17.53, 24.30, and 26.77 μg m^-2^ h^-1^ and 11.82, 17.30, 25.13, and 27.94 μg m^-2^ h^-1^ for cultivars T18 and T198, respectively. In 2020–2021, the highest fluxes were 6.32, 17.90, 27.67, and 31.03 μg m^-2^ h^-1^ and 6.51, 19.14, 26.61, and 31.19 μg m^-2^ h^-1^ for N0, N168, N240, and N312 of cultivars T18 and T198, respectively, when sown in CD. However, they were 6.20, 16.36, 26.67, and 28.65 μg m^-2^ h^-1^ and 6.54, 17.65, 25.64, and 28.51 μg m^-2^ h^-1^, respectively, for cultivars T18 and T198 when sown in WB. The flux gradually decreased following the peak pulse.

#### 3.2.2 Cumulative N_2_O emissions

**Figure 2:**
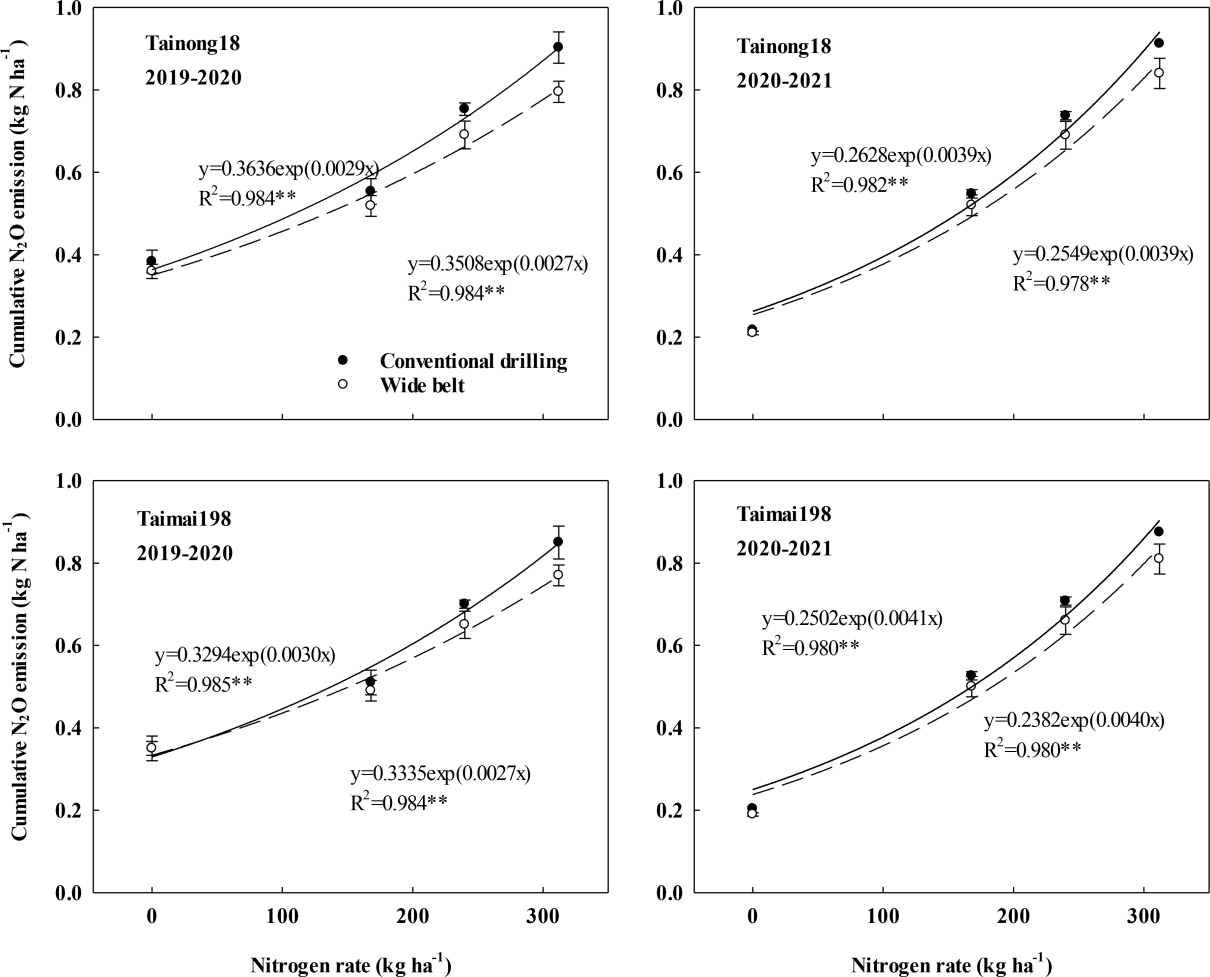
Effects of sowing patterns and N rates on cumulative nitrous oxide (N_2_O) emissions of winter wheat.

The cumulative N_2_O and GWP of N_2_O emissions were significantly influenced by the growing season, cultivar, N rate, and sowing pattern. Only the growing season × N rate and N rate × sowing pattern interactions significantly affected this measure (**Table S2**). The cumulative N_2_O emissions were 0.21–0.91 kg N ha^-1^ with an average of 0.60 kg N ha^-1^ for cultivar T18 and 0.19–0.87 kg N ha^-1^ with an average of 0.57 kg N ha^-1^ for cultivar T198, across two growing seasons (**Figure 2**). The GWP was 57.33–248.43 kg CO_2_-eq ha^-1^ with an average of 163.80 kg CO_2_-eq ha^-1^ for cultivar T18 and 51.87–237.51 kg CO_2_-eq ha^-1^ with an average of 155.61 kg CO_2_-eq ha^-1^ for cultivar T198, across two growing seasons (**Figure S3**).

Under each sowing pattern, the cumulative N_2_O emissions increased exponentially as the N rate increased from N0 to N312. WB significantly reduced the values at N168, N240, and N312 compared to CD and had a lower exponential equation slope (**Figure 2**), indicating that WB could slow the increase in N_2_O emissions resulting from the increased N rate. Therefore, the reduction in cumulative N_2_O emissions in WB significantly improved as the N rates increased and peaked at an N312. In 2019–2020, WB decreased the values by 6.29%, 8.31%, and 11.90%, and by 5.00%, 7.58%, and 9.61% for cultivars T18 and T198 at N168, N240, and N312, respectively. In 2020–2021, WB decreased the values by 4.69%, 7.07%, and 7.99%, and by 4.48%, 6.23%, and 7.64% for cultivars T18 and T198 at N168, N240, and N312, respectively. CD at N312 showed the highest cumulative N_2_O emissions. The GWP response of N_2_O emissions to sowing pattern and N rate showed the same trend as the cumulative N_2_O emissions (**Figure S3**).

**Figure 3:**
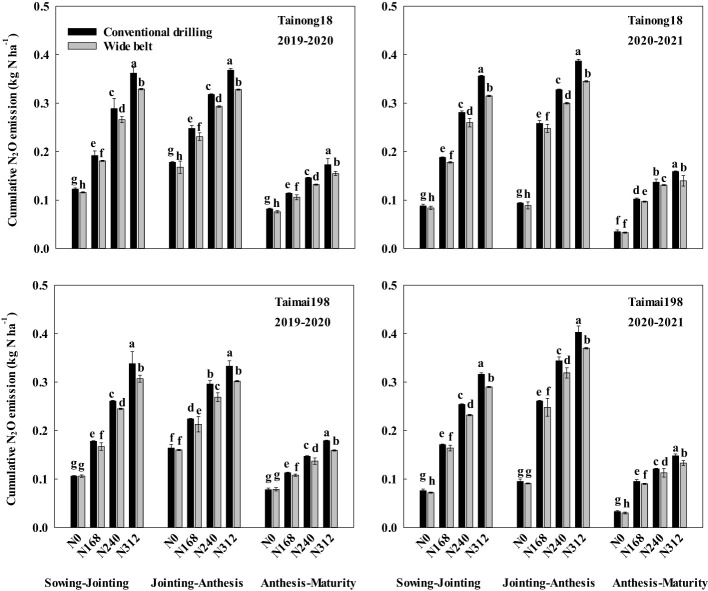
Effects of sowing patterns and N rates on cumulative nitrous oxide (N_2_O) emissions during different growth stages of winter wheat. N0, N168, N240, and N312 indicate N rates of 0, 168, 240, and 312 kg ha^-1^. Different letters within the same growth stage for the same season and cultivar indicate significant differences at *P* < 0.05.

As shown in **Figure 3** and **Table S3**, the cumulative N_2_O emissions during the stages from sowing to jointing, jointing to anthesis, and anthesis to maturity were 0.10–0.36, 0.13–0.38, and 0.05–0.17 kg N ha^-1^ with averages of 0.21, 0.27, and 0.11 kg N ha^-1^ for cultivar T18. For cultivar T198, these were 0.09–0.33, 0.13–0.37, and 0.05–0.16 kg N ha^-1^ with averages of 0.21, 0.26, and 0.11 kg N ha^-1^, respectively, across two growing seasons. The biggest proportion of cumulative N_2_O emissions was that from jointing to anthesis (39.22–47.44% with an average of 44.60%), followed by sowing to jointing (30.35–40.62% with an average of 36.32%) and anthesis to maturity (15.67–22.95% with an average of 19.08%).

The cumulative N_2_O emissions in WB during these three stages were all lower than in CD at N168, N240, and N312. Like the total cumulative N_2_O emissions, the amount and proportion of the reduced cumulative N_2_O emissions during the three stages also improved with increasing N rates and had the highest reduction at N312. The largest difference in N_2_O emissions for the two sowing patterns occurred from jointing to anthesis (0.011–0.043 kg N ha^-1^ with 0.025 kg N ha^-1^ on average), followed by sowing to jointing (0.007–0.041 kg N ha^-1^ with 0.021 kg N ha^-1^ on average), and anthesis to maturity (0.005–0.020 kg N ha^-1^ with 0.011 kg N ha^-1^ on average) across two growing seasons, for both cultivars and with N168, N240, and N312.

### 3.3 N_2_O emissions factors

**Figure 4:**
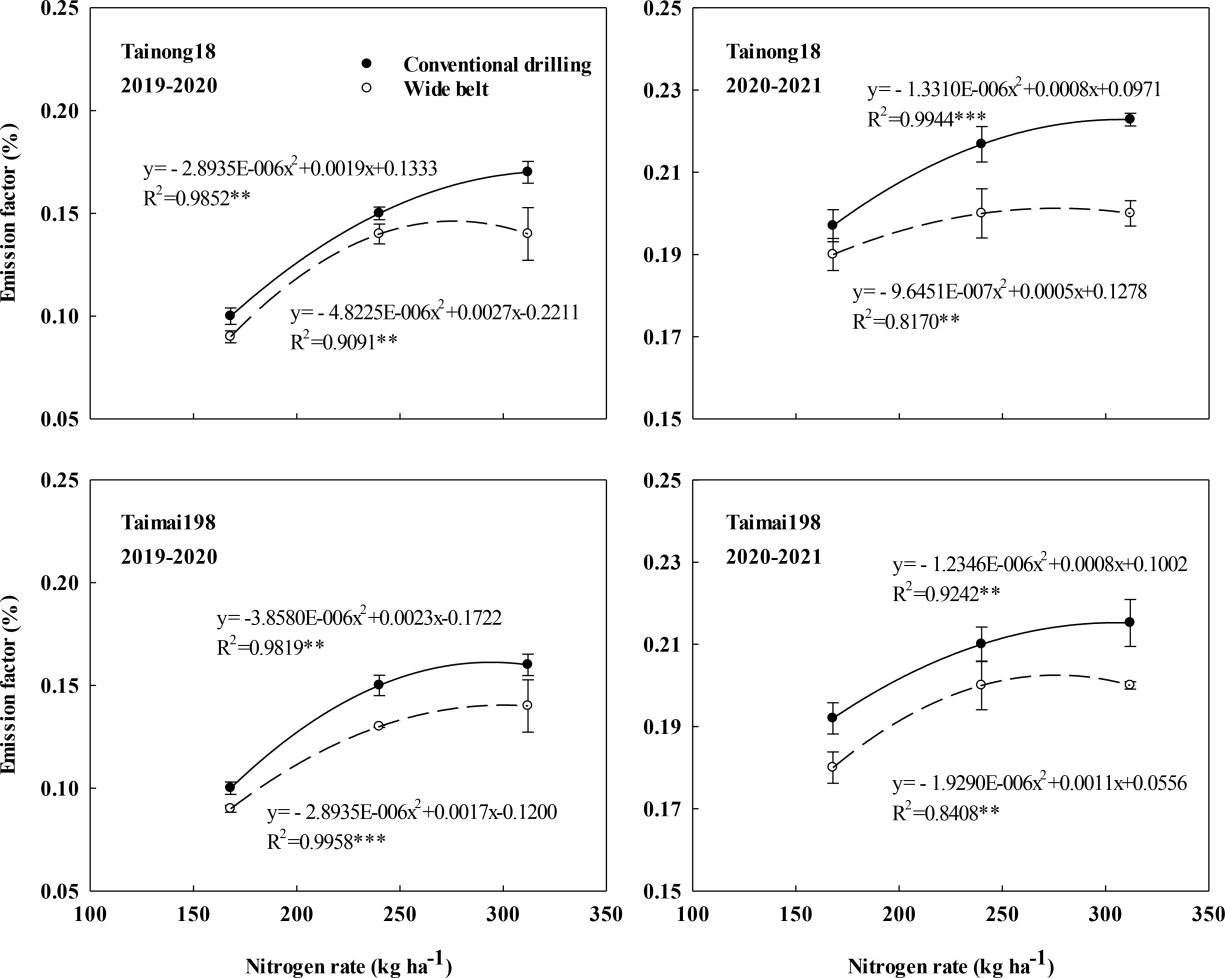
Effects of sowing patterns and N rates on the nitrous oxide emission factor of winter wheat.

The N_2_O EFs were significantly influenced by the main effects of growing seasons, cultivar, N rate, and sowing pattern. Only the interactions of growing season × N rate and N rate × sowing pattern significantly affected the N_2_O EFs (**Table S2**). The N_2_O EFs were 0.09–0.22% with an average of 0.17% for cultivar T18 and 0.09–0.22% with an average of 0.16% for cultivar T198 across two growing seasons (**Figure 4**). They were much higher in 2020–2021 (0.20%) than in 2019–2020 (0.13%), mainly due to the lower cumulative N_2_O emissions at N0 in the second growing season as a result of continuous not applying N fertilizer. Meanwhile, there was a parabolic change as the N rate increased from N168 to N312 under each sowing pattern.

The N_2_O EFs peaked at 293.20–319.00 kg ha^-1^ with an average of 305.63 kg ha^-1^ in CD, which was higher than that in WB (275.92–295.49 kg ha^-1^ with 283.55 kg ha^-1^ on average). WB significantly decreased the N_2_O EFs at N168, N240, and N312 compared to CD and showed a lower slope in the parabolic equation (**Figure 4**). Hence it reduced the increase in EFs resulting from the increased N rate. The extent of this reduction was significantly improved as the N rates increased and was highest at N312. In 2019–2020, WB decreased the EFs by 6.47%, 10.49%, and 16.09%, and by 13.66%, 14.16%, and 15.68% for cultivars T18 and T198 at N168, N240 and N312, respectively. In 2020–2021, WB decreased the values by 4.36%, 7.85%, and 8.86%, and by 4.19%, 6.76%, and 8.47% for cultivars T18 and T198 at N168, N240, and N312, respectively. CD at N312 had the highest N_2_O EFs. WB at N312 decreased the EFs of T18 and T198 by 9.40% and 8.53% in 2019–2020 and by 6.32% and 6.21% in 2020–2021, respectively, compared to the local management (sowing using CD at N240).

### 3.4 Yield-scaled N_2_O emissions

**Figure 5:**
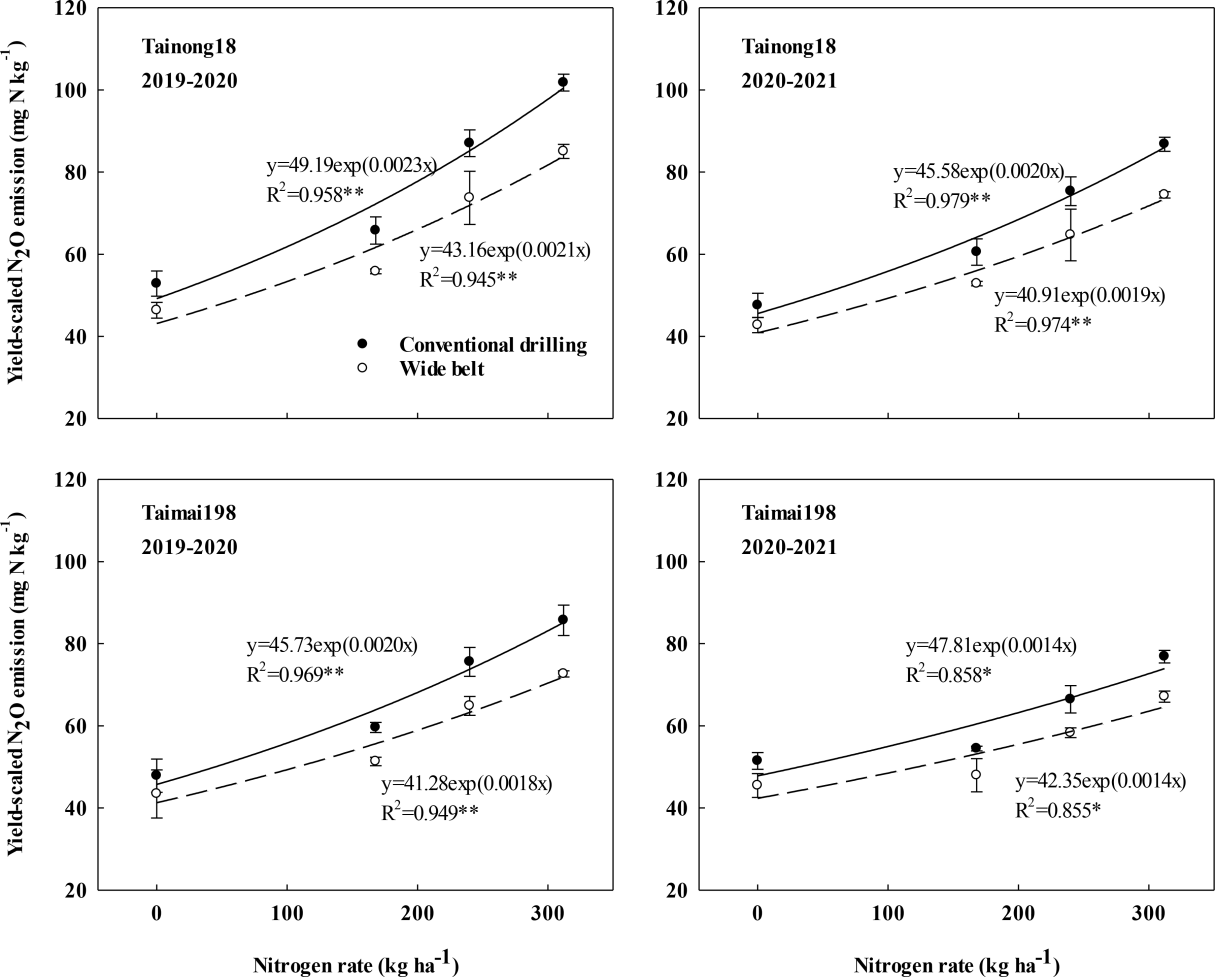
Effects of sowing pattern and N rate on yield-scaled nitrous oxide (N_2_O) emissions of winter wheat.

The yield-scaled N_2_O emissions were significantly influenced by the effects of the growing season, cultivar, N rate and sowing pattern, and the two-way interactions except for growing season × sowing pattern (**Table S2**). Across two growing seasons, the values were 42.77–101.78 mg kg^-1^ with 67.09 mg kg^-1^ on average for cultivar T18 and 43.41–85.69 mg kg^-1^ with 60.56 mg kg^-1^ on average for cultivar T198, respectively (**Figure 5**).

The yield-scaled N_2_O emissions increased exponentially as the N rate increased from N0 to N312 under the same sowing pattern. WB significantly reduced the values at each N rate compared to CD. The lower slope of the exponential equation indicated that WB could slow the increment of these emissions resulting from the increased N rate (**Figure 5**). Therefore, the reduction of yield-scaled N_2_O emissions in WB significantly improved as the N rates increased and were highest at N312. In 2019–2020, WB decreased this measure by 12.26%, 15.15%, 15.28%, and 16.44% and by 9.25%, 13.88%, 14.16%, and 15.23% for cultivars T18 and T198 at N0, N168, N240, and N312, respectively. In 2020–2021, the values decreased by 10.06%, 12.70%, 14.15%, and 14.19% and by 11.63%, 11.91%, 12.25%, and 12.66% for cultivars T18 and T198 at N0, N168, N240, and N312 kg ha^-1^, respectively. Furthermore, the reductions with seedling belt optimization were greater than those of cumulative N_2_O emissions. CD at N312 kg ha^-1^ obtained the highest yield-scaled N_2_O emissions. The emissions of WB at N312 were equal to the local management system (sowing with CD at N 240).

### 3.5 N uptake and soil inorganic N concentrations

#### 3.5.1 N uptake during different growth stages

**Figure 6:**
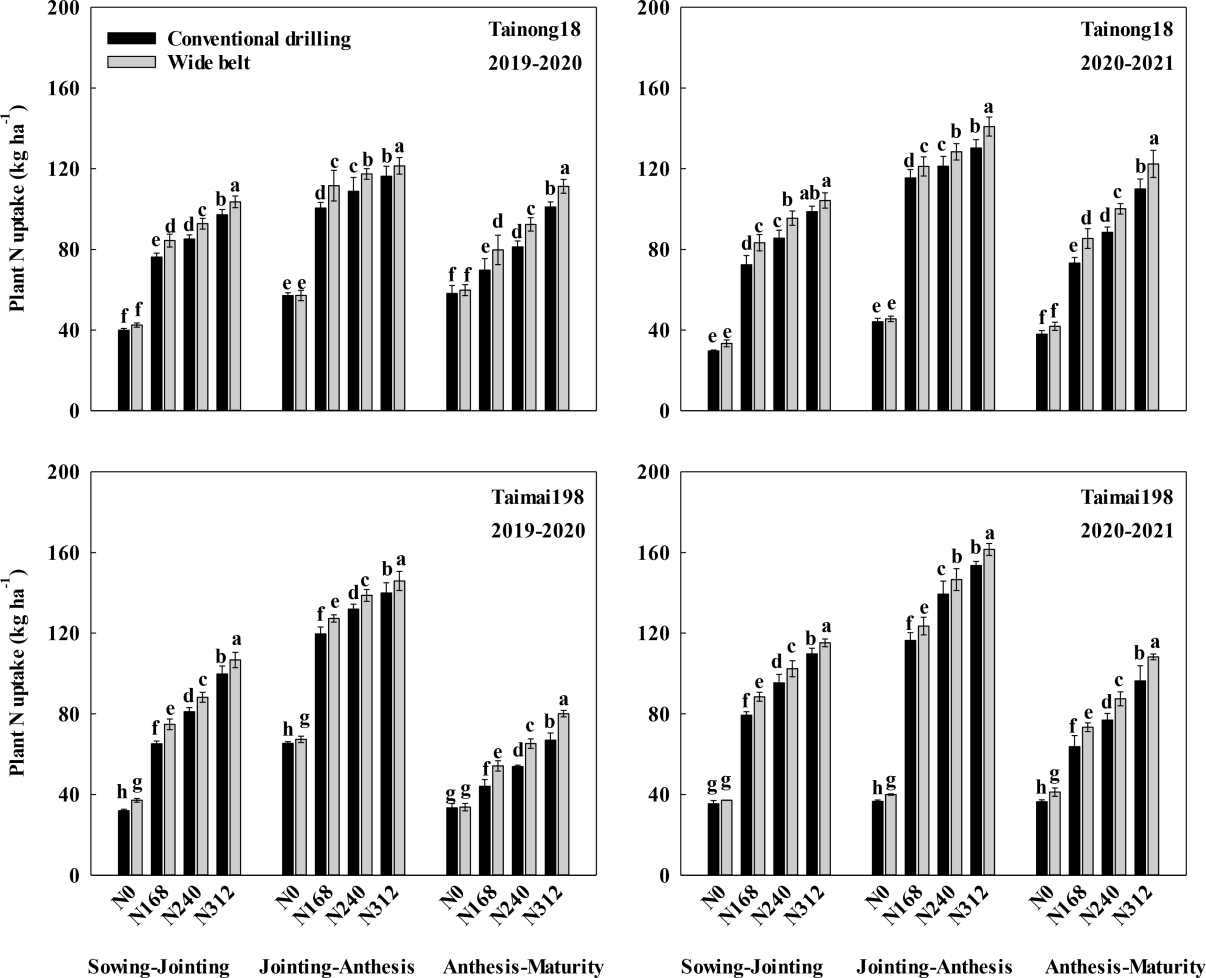
Effects of sowing pattern and N rate on plant N uptake during different winter wheat growth stages. N0, N168, N240, and N312 are N rates of 0, 168, 240, and 312 kg ha^-1^. Different letters within the same growth stage for the same season and cultivar indicate significant differences (*P* < 0.05).

The N uptake during the three growth stages increased with increasing N rates and peaked at N312 (**Figure 6**). The uptake during the three growth stages was higher in WB than in CD. At N168, N240, and N312, the differences in uptake for the two sowing patterns were 9.53, 8.74, and 5.99 kg ha^-1^ for T18 and 9.36, 7.06, and 6.22 kg ha^-1^ for T198 during sowing to jointing, 8.39, 7.90, and 7.93 kg ha^-1^ for T18 and 7.41, 6.99, and 6.97 kg ha^-1^ for T198 during jointing to anthesis, and 11.13, 11.39, and 11.33 kg ha^-1^ for T18 and 9.83, 10.94, and 12.52 kg ha^-1^ for T198 during anthesis to maturity, respectively, across two growing seasons.

#### 3.5.2 Soil inorganic N concentrations at different stages

**Figure 7:**
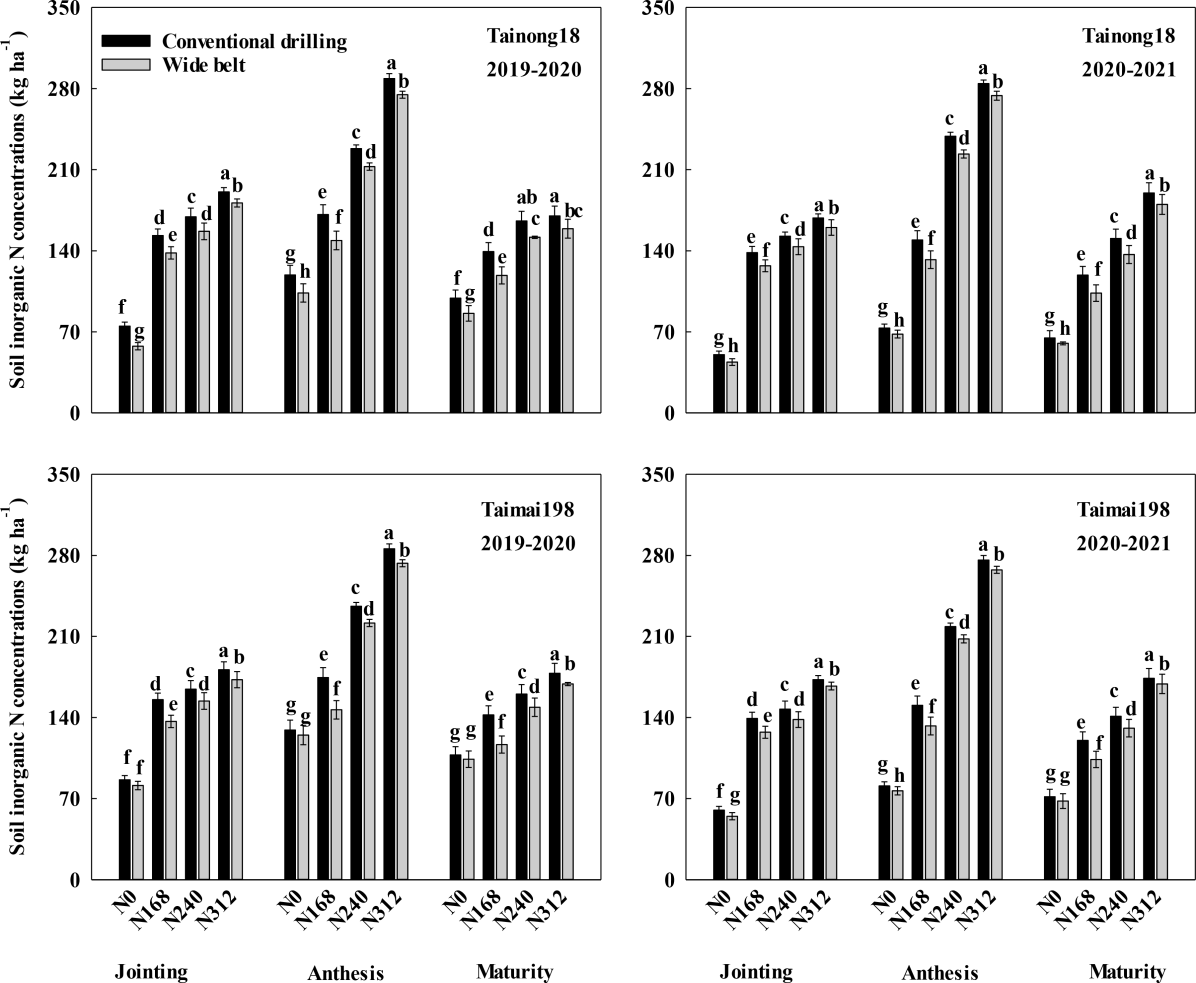
Effects of sowing pattern and N rate on soil inorganic N concentrations during different growth stages of winter wheat. N0, N168, N240, and N312 are N rates of 0, 168, 240, and 312 kg ha^-1^. Different letters within the same growth stage for the same season and cultivar indicate significant differences (*P* < 0.05).

The inorganic N concentrations in the soil at the three stages increased with increasing N rates and peaked at N312 (**Figure 7**). The concentrations at the three growth stages were lower in WB than in CD. At N168, N240, and N312, the differences were 13.18, 10.84, and 8.80 kg ha^-1^ for T18 and 15.31, 9.65, and 7.03 kg ha^-1^ for T198 at jointing, 19.63, 15.48, and 12.13 kg ha^-1^ for T18 and 22.81, 12.56, and 10.50 kg ha^-1^ for T198 at anthesis, and 18.07, 14.02, and 10.52 kg ha^-1^ for T18 and 21.00, 10.82, and 7.06 kg ha^-1^ for T198 at maturity, respectively, across two growing seasons.

### 3.6 Correlation analyses

We conducted correlation analyses between the cumulative N_2_O emissions and plant N uptake and soil inorganic N concentrations at N168, N240, and N312, respectively (**Table 2**). The N_2_O emissions were significantly negatively related to plant N uptake during the growth stages of sowing to jointing, jointing to anthesis, and anthesis to maturity. However, they were significantly positively related to the soil inorganic N concentrations at jointing, anthesis, and maturity for N168, N240, and N312.

**Table 2:** Results of correlation analyses of cumulative nitrous oxide emissions during different growth stages with plant N uptake and soil inorganic N concentrations.

N rate (kg ha^-1^)	Growth stage	Plant N uptake during growth stage	Soil inorganic N concentrations at the growth stage
168	Sowing-jointing/Jointing	-0.49*	0.47*
	Jointing-anthesis/Anthesis	-0.58**	0.41*
	Anthesis-maturity/Maturity	-0.72**	0.46*
240	Sowing-jointing/Jointing	-0.55**	0.46*
	Jointing-anthesis/Anthesis	-0.49*	0.41*
	Anthesis-maturity/Maturity	-0.61**	0.48*
312	Sowing-jointing/Jointing	-0.86**	0.46*
	Jointing-anthesis/Anthesis	-0.51**	0.51*
	Anthesis-maturity/Maturity	-0.73**	0.50*

* and ** indicate significance at P < 0.05 and 0.01, respectively.

## 4 Discussion

### 4.1 Influences of WB on grain yield and N uptake and utilization at different N rates

High wheat yields and NUE are based on plant N uptake (Duan et al., 2019). The arrangement of the wheat plants in the field may significantly affect growth and N uptake (Lu et al., 2020). WB sowing increased the belt of wheat seedlings and reduced the intraspecific competition of plants within the belts (Liu et al., 2020). This benefitted the growth of tillers and roots and resulted in an efficient N absorption capacity during the whole wheat growing season (Lv et al., 2020). In the present study, WB markedly improved grain yield and NUE at N168, N240, and N312, due to the improved plant N uptake, in line with Wang et al. (2022) and Zheng et al. (2023). This may be related to the improved activity of N assimilation enzymes in WB sowing, because higher activities of N assimilation enzymes, such as nitrate reductase, nitrite reductase, glutamine synthetase, glutamate synthase, are beneficial for crop N assimilation and absorption (Chandna et al., 2012; Agnihotri and Seth, 2016; Gupta and Seth, 2019). Furthermore, the combination of WB at N312 obtained the highest yield (most >11,000 kg ha^-1^), demonstrating that WB can be used to gain high grain yield at various N rates, especially at higher N rates.

### 4.2 Influences of WB on N_2_O emissions at different N rates

N_2_O emissions exhibited seasonal variation, and some studies have found that the cumulative N_2_O emissions at each winter wheat growing stage gradually decreased and were concentrated during the sowing to the greening stage (Liu et al., 2015). Nevertheless, Ji et al. (2012) demonstrated that they were higher from the greening to maturity stage than from the sowing to greening stage. This discrepancy may be due to the differences in specific basal/topdressing fertilizer ratios, temperature, rainfall, irrigation, or other factors. In our study, these emissions were concentrated at jointing to anthesis, followed by sowing to jointing, and anthesis to maturity. Furthermore, the reduced cumulative N_2_O emissions in the three growth stages followed a similar pattern in WB compared to CD. This was probably due to the 60% N topdressing (60% of total N fertilizer), irrigation at the jointing stage (60 mm), and suitable temperature (average 12.6°C) during the jointing to anthesis growth stage. These conditions favor soil nitrification and denitrification (Pan et al., 2022) and produce more N_2_O emissions.

N fertilization contributes to N_2_O emissions (Rahman et al., 2021). Despite a linear relationship between the cumulative N_2_O emissions and N rates (Kim et al., 2013), there is overwhelming evidence in the literature indicating that cumulative N_2_O emissions increase exponentially as the N rate increases, including evidence for grain crops around the world (Shcherbak et al., 2014), tropical sugarcane in Australia (Takeda et al., 2021), and spring wheat in New Mexico (Millar et al., 2018). Therefore, mitigation of N_2_O emissions at higher N input may be more difficult because some simple measures, such as supplementing with phosphate fertilizer (Shen and Zhu, 2022) and changing from conventional to no tillage (Campanha et al., 2019), mitigated N_2_O emissions at relatively low N input, but not at higher N rates. An exponential relationship was also found in the present study. Although the sowing pattern did not influence the exponential relationship between an increase in N_2_O emissions and increased N rates, WB initially decreased the N_2_O emissions for N168, N240, and N312 compared to CD and slowed the increase in cumulative N_2_O emissions resulting from the increased N rate. As a result, WB had greater N_2_O emissions at N312 compared to CD, indicating that WB could be used to reduce N_2_O emissions at various N rates, especially higher ones. A similar relationship was also observed between the GWP of N_2_O emissions and interactions of N rate and sowing pattern.

N_2_O EFs are used to estimate the direct N_2_O emissions in field crops, reflecting the differences in management patterns (Yue et al., 2019). The EFs in the present study were 0.09–0.22% when N rates increased from N168 to N312, like the reference values in Yan et al. (2015). Rahman et al. (2021) found that N_2_O EFs were linearly correlated with increasing N rates; however, other studies have demonstrated an exponential (Grace et al., 2016) or hyperbolic relationship (Kim et al., 2013) between EFs and N rates. Nevertheless, we observed a parabolic response, and the N rate at which N_2_O EFs theoretically peaked in WB (275.92–295.49 kg ha^-1^ with 283.55 kg ha^-1^ on average) was lower than that in CD (293.20–319.00 kg ha^-1^ with 305.63 kg ha^-1^ on average). This was probably due to the reduced cumulative N_2_O emissions in WB as the N rates increased.

### 4.3 N uptake, soil N concentrations, and their relationship with N_2_O emissions

Efficient N uptake and decreased concentrations of inorganic N in the soil may reduce N_2_O emissions (Xu et al., 2022). A review of 12 leading cultivars used in China’s major winter wheat cropping regions since the 1940s found that new wheat cultivars have a higher capacity to increase N uptake and reduce N_2_O emissions than older wheat cultivars (Ying et al., 2019). Chen et al. (2021) also demonstrated that new wheat cultivars reduce N_2_O emissions mainly through their higher productivity and N uptake and lower soil inorganic N availability. Similarly, the application of control-released fertilization also mitigates N_2_O emissions by maintaining the substrate content of inorganic N at a lower level in the soil than conventional fertilizers (Zhang et al., 2019).

At N168, N240, and N312, changing the sowing pattern from CD to WB markedly increased plant N uptake during the growth stages of sowing to jointing, jointing to anthesis, and anthesis to maturity and reduced the soil N concentrations at jointing, anthesis, and maturity. This was mainly due to WB-associated efficient N uptake capacity throughout the wheat growing season (Lv et al., 2020). Meanwhile, the cumulative N_2_O emissions during the different stages were significantly negatively related to the N uptake and positively related to the inorganic N concentrations in the soil, indicating that WB mitigated N_2_O emissions mainly through efficient N uptake and reduced soil inorganic N concentrations.

### 4.4 Influences of WB on yield-scaled N_2_O emissions at different N rates

N is an essential nutrient for crop production. Higher N fertilizer rates can often result in higher grain yields, resulting in a corresponding increase in N_2_O emissions (Millar et al., 2018). Unilaterally focusing on reducing the N_2_O emissions by reducing N rates may be counterproductive and lead to low grain yields (Duan et al., 2019). While meeting agricultural production needs, practices that minimize N_2_O emissions must be identified (Kim et al., 2022). Yield-scaled N_2_O emissions could be used as a benchmark to meet the critical global challenge of reducing N_2_O emissions while ensuring food security (Kong et al., 2021).

Field studies have reported an exponential increase between yield-scaled N_2_O emissions and increased N rates (Ma et al., 2010; Ji et al., 2012). We observed a similar relationship regardless of the sowing pattern, mainly due to the exponentially increased N_2_O emissions (R^2^ = 0.89, *P* < 0.01). Therefore, it is necessary to exploit efficient measures to reduce the yield-scaled N_2_O emissions when a specific N fertilizer is applied to achieve a high yield. New crop genotypes can increase plant N uptake (Chen et al., 2021), and other methods, such as new controlled-release fertilizers (Ji et al., 2012), partial substitution of chemical N with manure (Kong et al., 2021), and the addition of urease and nitrification inhibitors (Wang et al., 2021) can reduce the concentrations of inorganic N in the soil. All these measures would effectively decrease yield-scaled N_2_O emissions and improve yields. Meanwhile, in the present study, WB significantly reduced the yield-scaled N_2_O emissions at N168, N240, and N312 compared to CD due to the increased grain yield and reduced N_2_O emissions caused by the efficient N uptake and reduced soil N concentrations. Furthermore, we observed an increased reduction in yield-scaled N_2_O emissions in WB at high N rates, indicating that WB could play an important role in reducing such emissions when more synthetic N fertilizers are applied to in an effort to obtain high yields in the future. Besides, the yield-scaled N_2_O emissions were not significantly different between WB at N312 and the local management system (sowing using CD at N240); however, the grain yield was much higher. In conclusion, WB sowing can decrease N_2_O emissions and synergistically obtain high grain yields and NUEs, especially at higher N rates.

Although the application of higher N rates can improve the yield of winter wheat, it also reduces NUE and increases N_2_O emissions, regardless of sowing pattern. Moreover, higher N fertilizer application may decrease profits and increase carbon emissions considering the manufacture and transport of agricultural products. Therefore, more attention should be paid to the joint goals of increasing grain yield and reducing N rate. Combining WB sowing with the use of new controlled-release fertilizers or partial substitution of chemical N with manure may be efficient pathways to synergistically improve grain yield and reduce N input in the future.

## 5 Conclusions

WB sowing significantly increased the N uptake of winter wheat and reduced soil inorganic N concentrations, thereby markedly decreasing the cumulative N_2_O emissions, N_2_O EFs, GWP of N_2_O, and grain yield-scaled N_2_O emissions, and increased grain yield and NUE at N168, N240, and N312 compared to CD. Furthermore, these N_2_O emissions indices showed a larger reduction at higher N rates. Therefore, optimizing the seeding belt of wheat seedlings with high N rate input is an efficient way to mitigate greenhouse gases and improve yields and NUE. Our study shows the feasibility of attaining both high yield and low N_2_O emissions. However, more attention should be paid to the issues of increasing grain yield and reducing N rate in the future.

## Data availability statement

The original contributions presented in the study are included in the article/**Supplementary Material**. Further inquiries can be directed to the corresponding author.

## Author contributions

XD designed the experiments, managed the projects and guided the writing of the article. XZ and ML performed the experiments. XZ performed the data analysis and wrote the manuscript. FZ, YH and JC provided help on the experiments. YD and MH gave useful suggestions during the process of this experiments and article. All of authors listed have approved the manuscript that is enclosed.
