# Supplementary material for: Optimizing sowing patterns in winter wheat can reduce N2O emissions and improve grain yield and NUE by enhancing N uptake
Source: Front Plant Sci. 2023 May 31;14:1176293. doi: 10.3389/fpls.2023.1176293 (PMC10264632; doi:10.3389/fpls.2023.1176293)
Supplement: Supplementary file 1 [file DataSheet_1.docx]

**Supplementary material**

**Calculation formulas**

Plant N uptake (kg ha­^-1^) = AGN of the latter growth stage (kg ha­^-1^) - AGN of the previous growth stage (kg ha­^-1^) (1)

NUE (kg kg­^-1^) = grain dry weight / N available (2)

UPE (%) = AGN / N available × 100 (3)

UTE (kg kg­^-1^) = grain dry weight / AGN (4)

F = 273/ (273 + T) × 28/22.4 × 60 × H × dc/dt (5)

where F is the N_2_O emissions flux (μg N_2_O-N m^-2^ h^-1^), T is the temperature in the chamber (°C), 28 is the atomic mass of N in N_2_O, 22.4 is the molar volume of N_2_O at a temperature of 273 K, H is the height of the sampling box (cm), c is the concentration of N_2_O gas (μL L^-1^), t is the closing time (min), and dc/dt is the change rate of N_2_O gas concentration in the sampling box (μL L^-1^ min^-1^).

E(N_2_O) = ∑[(F_i+1_ + F_i_)/2] × (D_i+1_-D_i_) × 24/1000 (6)

where E(N_2_O) is the cumulative N_2_O emissions (mg N m^-2^), F_i_ and F_i+1_ are the N_2_O fluxes at the ith and i+1th measurements (μg N m^-2^ h^-1^), respectively, and D_i_ and D_i+1_ are the times at the ith and i+1th measurements (d).

EF (%) = [E_N_(N_2_O) - E_0_(N_2_O)]/N × 100 (7)

where E_N_(N_2_O) is the cumulative N_2_O emissions with N fertilizer input and E_0_(N_2_O) is the cumulative N_2_O emissions without N fertilizer input, and N is the amount of N rate applied.

N_2_O GWP (kg N ha^-1^) = cumulative N_2_O emissions × 273 (8)

where the number 273 corresponds to the IPCC factors for converting N_2_O into its CO_2_ equivalent.

Yield-scaled N_2_O (mg N kg^-1^) = cumulative N_2_O emissions/grain yield (9)

**Figures and tables**

Fig. S1. Rainfall and mean temperature were recorded during the winter wheat growth period (October to June) in the 2019–2020 and 2020–2021 growing seasons.

**Row spacing (25 cm)**

**Wide belt sowing (WB)**

**17 cm**

**Seeding belt width (8 cm)**

**Conventional drilling sowing (CD)**

**22 cm**

**Seedling belt width (3 cm)**

**Row spacing (25 cm)**


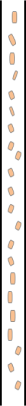

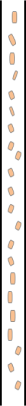

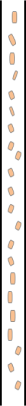

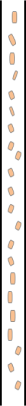


Fig. S2. Schematic diagram of conventional drilling sowing and wide belt sowing.

Fig. S3. The effects of sowing patterns and N rates on the global warming potential (GWP) of nitrous oxide in winter wheat.

Table S1. The fertilization scheme in winter wheat.

| Growing season | Fertilizer stage | Fertilizer application rate | | |
| --- | --- | --- | --- | --- |
|  |  | N (urea) | P (P_2_O_5_) | K (K_2_O) |
| 2019-2020 | Before sowing | 40% of the N fertilizer at each N rate | 120 kg ha^-1^ | 120 kg ha^-1^ |
|  | Jointing | 60% of the N fertilizer at each N rate | - | - |
| 2020-2021 | Before sowing | 40% of the N fertilizer at each N rate | 120 kg ha^-1^ | 120 kg ha^-1^ |
|  | Jointing | 60% of the N fertilizer at each N rate | - | - |

Table S2. Variance analysis of the effects of cultivar, growing season, nitrogen rate and sowing pattern on grain yield, nitrogen use efficiency (NUE), cumulative N_2_O emission, emission factor, global warming potential, yield-scaled N_2_O.

| Factor | Grain yield | NUE | Cumulative N_2_O emission | Emission factor | Global warming potential | Yield-scaled N_2_O emission |
| --- | --- | --- | --- | --- | --- | --- |
| Growing season (G) | 6.79* | 858.97*** | 34.24*** | 1893.31*** | 76.70*** | 86.36*** |
| Cultivar (C) | 92.98*** | 364.41*** | 34.83*** | 12.69*** | 79.26*** | 101.63*** |
| Nitrogen rate(N) | 1689.34*** | 17528.50*** | 2285.50*** | 191.69*** | 5130.67*** | 547.68*** |
| Sowing pattern (P) | 180.67*** | 1993.36*** | 72.25*** | 94.95*** | 167.15*** | 205.81*** |
| G×C | 1.41 | 68.76*** | 1.44 | 0.03 | 3.39 | 8.67** |
| G×N | 502.87*** | 9788.14*** | 69.76*** | 46.71*** | 1.51 | 11.06*** |
| G×P | 0.21 | 2.17 | 0.8 | 1.37 | 2.32 | 2.74 |
| C×N | 28.36*** | 327.37*** | 0.69 | 0.08 | 153.68*** | 13.75*** |
| C×P | 0.08 | 1.93 | 0.93 | 0.07 | 2.53 | 2.13* |
| N×P | 3.31* | 47.56*** | 8.82*** | 5.37** | 20.71*** | 6.32*** |
| G×C×N | 5.85** | 196.86*** | 0.06 | 0.19 | 0.17 | 1.61 |
| G×C×P | 0.41 | 2.81 | 0.43 | 0.57 | 0.77 | 0.22 |
| G×N×P | 0.98 | 24.43*** | 0.24 | 0.23 | 0.10 | 0.37 |
| C×N×P | 0.11 | 2.32 | 0.04 | 0.17 | 0.52 | 0.21 |
| G×C×N×P | 0.08 | 0.29 | 0.05 | 0.14 | 0.12 | 0.07 |

Note: *, ** and *** indicate significance at *P* < 0.05, 0.01, and 0.001, respectively.

Table S3. The effects of sowing patterns and N rates on ratio of cumulative nitrous oxide (N_2_O) emissions during the growth stages from sowing to jointing (S–J), jointing to anthesis (J–A), and anthesis to maturity (A–M) of winter wheat.

| Growing season | Cultivar | N rates | Sowing pattern | S-J | J-A | A-M |
| --- | --- | --- | --- | --- | --- | --- |
|  |  | (kg ha^-1^) |  | (%) | (%) | (%) |
| 2019-2020 | Tainong18 | 0 | Conventional drilling | 32.18d | 46.46a | 21.36a |
|  |  |  | Wide belt | 32.12d | 46.72a | 21.16a |
|  |  | 168 | Conventional drilling | 34.67c | 44.72b | 20.60b |
|  |  |  | Wide belt | 35.00c | 44.47b | 20.54b |
|  |  | 240 | Conventional drilling | 38.36b | 42.23c | 19.41c |
|  |  |  | Wide belt | 38.53b | 42.42c | 19.05c |
|  |  | 312 | Conventional drilling | 40.10a | 40.77d | 19.13c |
|  |  |  | Wide belt | 40.45a | 40.42d | 19.13c |
|  | Taimai198 | 0 | Conventional drilling | 30.35d | 47.16a | 22.49ab |
|  |  |  | Wide belt | 30.70d | 46.35a | 22.95a |
|  |  | 168 | Conventional drilling | 34.51c | 43.53b | 21.95b |
|  |  |  | Wide belt | 34.26c | 43.67b | 22.07b |
|  |  | 240 | Conventional drilling | 37.09b | 42.07c | 20.84c |
|  |  |  | Wide belt | 37.63b | 41.33c | 21.07c |
|  |  | 312 | Conventional drilling | 39.73a | 39.22d | 21.06c |
|  |  |  | Wide belt | 39.95a | 39.30d | 20.75c |
| 2020-2021 | Tainong18 | 0 | Conventional drilling | 40.56a | 43.33b | 16.11c |
|  |  |  | Wide belt | 40.62a | 43.19b | 16.19c |
|  |  | 168 | Conventional drilling | 34.25c | 47.18a | 18.57a |
|  |  |  | Wide belt | 34.01c | 47.44a | 18.54a |
|  |  | 240 | Conventional drilling | 37.64b | 43.94b | 18.42a |
|  |  |  | Wide belt | 37.66b | 43.42b | 18.91a |
|  |  | 312 | Conventional drilling | 39.46a | 42.91b | 17.63b |
|  |  |  | Wide belt | 39.39a | 43.13b | 17.48b |
|  | Taimai198 | 0 | Conventional drilling | 37.34a | 46.67b | 15.99c |
|  |  |  | Wide belt | 37.17a | 47.17b | 15.67c |
|  |  | 168 | Conventional drilling | 32.47c | 49.54a | 17.99a |
|  |  |  | Wide belt | 32.70c | 49.37a | 17.93a |
|  |  | 240 | Conventional drilling | 35.30b | 47.93b | 16.77bc |
|  |  |  | Wide belt | 35.00b | 47.99b | 17.01b |
|  |  | 312 | Conventional drilling | 36.40a | 46.49b | 17.11b |
|  |  |  | Wide belt | 36.54a | 46.65b | 16.81bc |

Note: Different letters within a column for the same season and cultivar indicate significant difference (*P* < 0.05).
